# Chondrogenic and Osteogenic In Vitro Differentiation Performance of Unsorted and Sorted CD34^+^, CD146^+^, and CD271^+^ Stem Cells Derived from Microfragmented Adipose Tissue of Patients with Knee Osteoarthritis

**DOI:** 10.3390/jcm14041184

**Published:** 2025-02-11

**Authors:** Jasmin Bagge, Haider Mahmood, Jennifer Janes, Kilian Vomstein, Lars Blønd, Lisbet R. Hölmich, Kristine Freude, Jan O. Nehlin, Kristoffer W. Barfod, Per Hölmich

**Affiliations:** 1 Sports Orthopedic Research Center—Copenhagen (SORC-C), Department of Orthopedic Surgery, Copenhagen University Hospital—Amager and Hvidovre, Kettegård Allé 30, 2650 Hvidovre, Denmark; haidermahmoodhaider@hotmail.com (H.M.); kristoffer.weisskirchner.barfod@regionh.dk (K.W.B.); per.hoelmich@regionh.dk (P.H.); 2 Veterinary Diagnostic Laboratory, University of Kentucky, 1490 Bull Lea Rd, Lexington, KY 40511, USA; jennifer.janes@uky.edu; 3 Department of Obstetrics and Gynecology, The Fertility Clinic, Copenhagen University Hospital—Hvidovre, Kettegård Allé 30, 2650 Hvidovre, Denmark; thomas.kilian.vomstein@regionh.dk; 4 Department of Orthopedic Surgery, Zealand University Hospital—Køge, Lykkebækvej 1, 4600 Køge, Denmark; lars-blond@dadlnet.dk; 5 Department of Plastic Surgery, Copenhagen University Hospital—Herlev and Gentofte, Borgmester Ib Juuls Vej 1, 2730 Herlev, Denmark; lisbet.rosenkrantz.hoelmich@regionh.dk; 6 Disease Stem Cell Models and Embryology, Department of Veterinary and Animal Sciences, University of Copenhagen, Grønnegårdsvej 7, 1870 Frederiksberg C, Denmark; kkf@sund.ku.dk; 7 Department of Clinical Research, Copenhagen University Hospital—Amager and Hvidovre, Kettegård Allé 30, 2650 Hvidovre, Denmark; jan.nehlin@regionh.dk; 8 Section of Sports Traumatology, Department of Orthopedic Surgery, Copenhagen University Hospital—Bispebjerg, Bispebjerg Bakke 23, 2400 København, Denmark

**Keywords:** microfragmented adipose tissue, magnetic-activated cell sorting, CD34^+^ stem cells, CD146^+^ stem cells, CD271^+^ stem cells, pericytes, osteoarthritis, differentiation potential

## Abstract

**Background/Objectives**: Treatment of knee osteoarthritis (OA) with autologous stem cells from microfragmented adipose tissue (MFAT) has shown promising but varying results. Multiple stem cell types, including CD34^+^, CD146^+^, and CD271^+^ stem cells, have been identified within MFAT. Patient-specific heterogeneity in stem cell populations and the content of highly potent cells may be determining factors for a successful treatment outcome. The current study aimed to identify the most promising stem cell type in MFAT to treat OA, focusing on their chondrogenic and osteogenic differentiation performance. **Methods:** CD34^+^, CD146^+^, and CD271^+^ stem cells from the MFAT of eight patients with knee OA were separated using magnetic-activated cell sorting (MACS) and analyzed as subtypes. Unsorted cells were used as a control. Chondrogenic and osteogenic in vitro differentiation were assessed through Safranin-O and H&E staining, pellet size, and qPCR for chondrogenesis, as well as Alizarin Red S staining and qPCR for osteogenesis. **Results:** CD34^+^, CD146^+^, and CD271^+^ stem cells were doubled using MACS. All subtypes were able to undergo osteogenic differentiation with Alizarin Red S staining, revealing a significant increase in calcium deposits of induced cells compared to non-induced controls. CD146^+^ stem cells showed higher calcium deposition compared to CD34^+^, CD271^+^, and unsorted stem cells. All cell types could form chondrogenic pellets. CD271^+^ stem cells produced more proteoglycans, as shown by Safranin-O staining, than CD34^+^ and CD146^+^ stem cells, but not more than the unsorted stem cells. After differentiation induction, all cell types showed an upregulation of most chondrogenic and osteogenic biomarkers. **Conclusions:** CD146^+^ stem cells showed the highest osteogenic differentiation performance for calcium deposition, while CD271^+^ stem cells showed the greatest chondrogenic differentiation performance for proteoglycan formation. The prevalence of these stem cell types may play a critical role in the clinical effectiveness when treating OA.

## 1. Introduction

Osteoarthritis (OA) is an inflammatory and degenerative joint disease causing progressive cartilage break-down and damage to the underlying bone. OA is a major cause of disability and affects more than 595 million people globally, with the knee being the most affected site. Due to the demographic rise in age and obesity, the number of patients with OA is believed to increase [1]. Articular cartilage injuries are particularly problematic since cartilage has a very limited intrinsic repair capacity [2]. Consequently, there is a great need for joint restoration methods. Unfortunately, no effective regenerative therapy exists in clinical practice. Conventional treatment includes physiotherapy, pain killers, braces, and, in end-stage OA, joint replacement [3]. Treatment of mild-to-moderate knee OA with autologous stem cells from microfragmented adipose tissue (MFAT) has, however, shown promising results with improved patient-reported outcome measurements, less OA progression, and some cartilage regeneration on imaging, but the results are varied [4,5,6]. Employing MFAT is a one-step procedure used to mechanically process stem cells for therapy directly in the operation theatre without the use of enzymes and is being increasingly used worldwide [7]. Multiple stem cell types have been identified in MFAT, such as CD34^+^, CD146^+^, and CD271^+^ stem cells, and the amount of each subtype varies greatly between patients [8,9,10]. CD34^+^ stem cells, from the perivascular niche of MFAT, are characteristic of adventitial stem cells ((ASCs) − CD31^−^/CD45^−^/CD34^+^/CD90^+^/CD146^−^) surrounding larger arteries and veins. CD146 is a heterophilic cell–cell adhesion molecule [11]. CD146^+^ stem cells, from the perivascular niche of MFAT, are characteristic of pericytes (CD31^−^/CD45^−^/CD34^−^/CD90^+^/CD90^+^/CD146^+^) which surround capillaries and micro vesicles [12,13]. CD271^+^ stem cells (CD31^−^/CD45^−^/CD90^+^/CD271^+^) have recently been identified in the inner region of the perivascular wall of adipose tissue and have previously been extensively studied in bone marrow [14,15]. ASCs and pericytes are identified as in vivo progenitors of cultured mesenchymal stem cells (MSCs) [10]. These subtypes exhibit varying differentiation capacities when derived from bone marrow or enzymatically processed adipose tissue [11,14,16]. However, comparative analysis of MFAT-derived cells is yet to be conducted. Of clinical interest are CD146^+^ stem cells, which have been reported to display a better osteogenic performance compared to MSCs and CD34^+^ stem cells when derived from bone marrow and enzymatically treated adipose tissue [13,17]. Importantly, CD146^+^ cells are enriched in MFAT compared to enzymatically processed adipose tissue, with distinct cellular profiles [10]. Of similar interest for clinical applications are CD271^+^ stem cells, which, when derived from enzymatically processed adipose tissue, have been shown to enhance cartilage repair and reduce angiogenesis compared to MSCs in a rat osteochondral defect model [15].

It is a well-known characteristic that stem cell potency and numbers can vary between individuals. Therefore, patient-dependent heterogeneity of stem cell populations and the content of highly potent cells may be determining factors for a successful treatment outcome [18]. Knowledge of the amount of potent stem cell pools based on a small adipose tissue biopsy can be used to identify suitable patients for MFAT treatment in a personalized manner. Moreover, separation and enrichment of potent subtypes and elimination of non-effective cells by new sorting methods may be a way to improve the clinical outcome. Magnetic-activated cell sorting (MACS) is a straightforward enrichment method for cells with specific surface markers, making it particularly suitable for clinical use, as all consumables are sterile and designed for single use.

The aim of this study was thus to identify the most promising stem cell type in MFAT to treat OA, focusing on their chondrogenic and osteogenic differentiation performance. The specific aims were as follows: (1) assess if CD34^+^, CD146^+^, and/or CD271^+^ stem cells derived from MFAT have a better chondrogenic and/or osteogenic in vitro differentiation performance compared to unsorted stem cells (representative of MFAT treatment) from MFAT, and (2) identify if one of the subtypes has a greater chondrogenic and/or osteogenic in vitro differentiation performance compared to the other subtypes.

## 2. Materials and Methods

### 2.1. Primary Cell Lines

MFAT from 8 patients with knee OA classified as Kellgren–Lawrence grade 2–3 (5 females, 3 males; Age 47.8 ± 9.2 years) was harvested in a randomized controlled trial with autologous MFAT treatment of mild–moderate knee OA (ClinicalTrials.gov Identifier: NCT03771989) [19]. Subcutaneous abdominal adipose tissue was harvested by lipoaspiration under local analgesia, followed by microfragmentation [19]. Stem cells were isolated in the laboratory by tissue explant culture (without enzymes) immediately after harvest, as described previously [9]. In short, 2 mL MFAT was transferred to two T75 cm^2^ culture flasks (TPP, Buch & Holm A/S, Herlev, Denmark), with 1 mL/T75 used for tissue explant culture at 37 °C in a humidified atmosphere containing 5% CO_2_. A total of 11 mL 37 °C expansion medium containing Dulbecco’s Modified Eagle Medium (DMEM, Gibco, 1 g/L glucose, Thermo Fisher Scientific, Waltham, MA, USA), 10% heat-inactivated fetal bovine serum (FBS, Gibco, Thermo Fisher Scientific), and 1% penicillin–streptomycin (P/S, Gibco, Thermo Fisher Scientific) were added per T75. The first medium change occurred after 48 h, where MFAT clusters and non-adherent cells were removed by aspiration and washed with Dulbecco’s phosphate-buffered saline (dPBS, Gibco, without calcium and magnesium, Thermo Fisher Scientific). At ~70% confluency, the cells were passaged with 0.25% Trypsin/1 mM EDTA (Gibco, Thermo Fisher Scientific). The cells were grown in T75 culture flasks at a seeding density of 500,000 cells/T75 and cultured with 11 mL expansion medium. The cells were cryopreserved at passage 1, thawed, and expanded [9] to passage 3 where they were analyzed by flow cytometry and sorted via MACS. Non-sorted cells were used as a control at passage 6 compared to sorted cells (paired samples) for differentiation analyses. Differentiation assays were performed at passage 6.

### 2.2. Magnetic-Activated Cell Sorting (MACS)

Plastic adherent passage 3 cells were counted using a Fast-Read^®^ 102 (VWR, Radnor, PA, USA) and trypan blue staining (Gibco, Thermo Fisher Scientific). CD34^+^, CD146^+^, and CD271^+^ stem cells were enriched using CD34, CD146, and CD271 Human MicroBead Kits (Miltenyi Biotech, Bergisch Gladbach, Germany), LS columns (Miltenyi Biotech), and a QuadroMACS Separator as per the manufacturer’s instructions (Miltenyi Biotech). For CD34^+^ and CD146^+^ cell sorting, 4.5 × 10^6^ viable cells were used for each. For CD271^+^ cell sorting, 6 × 10^6^ viable cells were used. In short, the cells were resuspended in MACS buffer (MACS bovine serum albumin stock solution) (Miltenyi Biotech) diluted 1:20 in autoMACS Rinsing Solution (Miltenyi Biotech). Fridge-cold FcR Blocking Reagent from the kits was added to minimize unspecific labeling. The cells were magnetically labeled with CD34, CD146, or CD271 microbeads and incubated in the dark for 15 min at 4 °C. The cells were washed with MACS buffer and centrifuged at 300× *g* for 10 min. The cell pellet was resuspended in MACS buffer and LS columns were prepared through MACS buffer rinsing. Labeled cells were positively selected in LS columns and separated by the magnetic field of the QuadroMACS Separator after washing the column thrice. Labeled cells were collected in a 15 mL conical tube by adding 37 °C pre-warmed expansion medium and firmly pushing the plunger into the column. Enriched cells were cultured in T75 with expansion medium at 37 °C and 5% CO_2_. The first medium change occurred after 48 h and then twice a week. MACS efficiency was assessed by flow cytometry. Three cell lines (one CD34^+^, one CD146^+^, and one CD271^+^) from different patients had to be excluded due to low proliferation after MACS (final *n* = 8 patients, N = 29 cell lines).

### 2.3. Flow Cytometry

**Figure 1:**
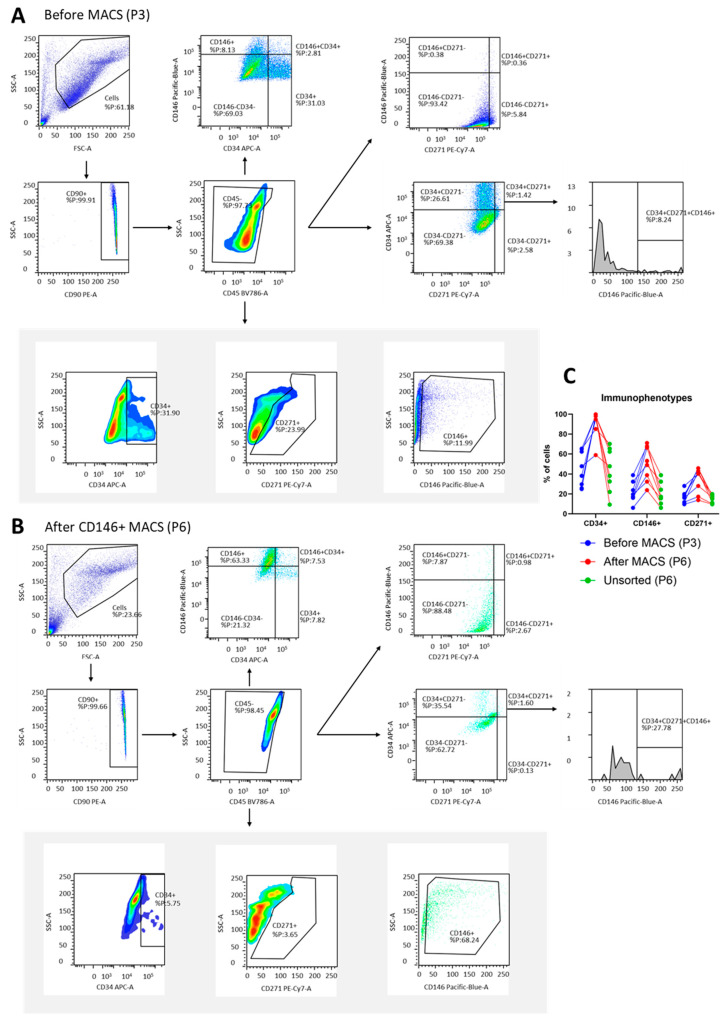
Immunophenotyping and gating strategy. (**A**) Flow cytometry gating strategy before magnetic-activated cell sorting (MACS). Representative dot plots and histograms depict gating sequence used to identify cell populations based on forward-scatter (FSC) and side-scatter (SSC) profiles. Initial gating was performed by exclusion of debris and selection of viable cells, followed by selecting CD90^+^ and CD45^−^ cells. Hereafter, CD34, CD146, and CD271 cell populations were identified. (**B**) Flow cytometry gating strategy after MACS for CD146^+^ stem cells. Representative dot plots and histograms illustrate refined gating strategy applied after CD146^+^ MACS, which show enrichment of CD146^+^ stem cells after sorting. (**C**) Percentage of immunophenotypes before and after MACS with lines connecting paired samples. Graph displays proportion of cells expressing CD34, CD146, and CD271 on cell surface before MACS (passage 3, P3, blue), after MACS (passage 6, P6, red), and in unsorted samples (P6, green). Results illustrate changes in surface marker expression following MACS and shows unsorted P6 as a control.

The immunophenotype of the cells was assessed by flow cytometry (BD LSR Fortessa with BD FACSDiva 8.0.3 software, BD Biosciences, Franklin Lakes, NJ, USA) at passage 3 (prior to MACS) and at passage 6 (immediately before assays). Multicolor flow cytometry and gating were carried out as previously described by our group [8,9] using multiple anti-human fluorescent primary antibodies, CD31-FITC, CD34-APC, CD45-BV786, CD90-PE, CD146-BV421, and CD271-PE-CyTM7 (BD, Horizon subtype, mouse IgG1κ isotype, BD Biosciences), in a 1:100 dilution. Additional gating for co-expression was performed based on the CD90^+^/CD45^−^ fraction, whereafter cells were gated for CD34^+^/CD146^+^, CD34^+^/CD271^+^, and CD146^+^/CD271^+^. Lastly, CD34^+^/CD271^+^ cells were gated for co-expression of CD146^+^ (CD34^+^/CD271^+^/CD146^+^) (Figure 1). Non-stained cells, fluorescence-minus-one (FMO), and mouse IgG_1_κ isotypes (BD Biosciences) for each fluorophore were used as controls and to optimize gating. BD FACSDiva™ CS&T Research Beads (BD Biosciences) were used to check flow cytometer performance prior to each assay.

### 2.4. Chondrogenic Differentiation

Passage 6 cells were induced to undergo chondrogenic differentiation as 3D pellets, as previously described [20]. A total of 500,000 cells were resuspended in 1 mL chondrogenic induction medium consisting of DMEM (4.5 g/L glucose, Gibco, Thermo Fisher Scientific), 1% P/S, 12.5 mg/mL bovine serum albumin (BSA, Sigma-Aldrich, St. Louis, MO, USA), 1× insulin-transferrin-selenium-sodium pyruvate growth supplement (Gibco, Thermo Fisher Scientific), 1× MEM non-essential amino acids (Gibco, Thermo Fisher Scientific), 100 nM dexamethasone (Sigma-Aldrich), 50 µg/mL L-ascorbic acid-2-phosphate sesquimagnesium salt hydrate (A2P, Sigma-Aldrich), and 10 ng/mL human recombinant transforming growth factor β_1_ (TGF-β_1_, Sigma-Aldrich). Pellets were generated by transferring the cell suspension to a vented 1.5 mL polypropylene tube and spinning at 500× *g* for 3 min. A total of 10 pellets were generated per cell line for each biological replicate. After 48 h, the pellets were transferred to a 1% poly 2-hydroxyethyl methacrylate (Sigma-Aldrich)-coated 24-well plate (TPP, Buch & Holm A/S, Herlev, Denmark) with 1 pellet per well to avoid cell adherence. After transfer, 1 mL fresh chondrogenic induction medium was added to each pellet. The pellets were cultured for 21 days at 37 °C and 5% CO_2_ in chondrogenic induction medium with medium change twice a week. As a control for gene expression analyses, non-induced passage 6 cells were grown as monolayers in T75 with expansion medium until being harvested at ~70% confluency for RNA isolation with one T75 per cell line.

### 2.5. Chondrogenic Pellet Size

After 21 days of chondrogenic induction, pellets were carefully transferred to a 24-well plate, with one pellet in 0.5 mL dPBS per well. Standardized 2D brightfield images of all pellets were captured at 4× magnification using a Leica DMIL LED fluorescent microscope (Triolab, Brøndby, Denmark). The shortest and longest side of the pellet was measured using Leica LAS X software (version 5.2.0.26130). Measurements were averaged per pellet and then per cell line.

### 2.6. Histological Assessments of Chondrogenic Pellets

Four randomly selected pellets per cell line were fixed in 4% paraformaldehyde (Pierce, in dPBS, Thermo Fisher Scientific) for 24 h at 4 °C and pre-embedded in 2% (*w*/*v*) BD Bacto^TM^ agar (Thermo Fisher Scientific)/2.5% (*w*/*v*) gelatin (type A, Sigma-Aldrich) with two pellets per block. The blocks were fixed in 4% paraformaldehyde for 24 h at 4 °C and placed in 70% ethanol at 4 °C for batched histological paraffinization. Pellets were sectioned at 5 µm at the center, placed on SuperFrost Plus adhesion glass slides (VWR), heat-fixed at 60 °C for 30 min, and stained as previously described [20] with 0.1% Safranin-O for 5 min to assess proteoglycan content and with Hematoxylin and Eosin (H&E) to evaluate cell morphology and pellet architecture. All sections were stained on the same day in 30-slide racks using the same batch of staining. Mature equine articular cartilage [20] was used as a calibrator with three randomly placed slides in each rack. Equine chondrocytes and dermal fibroblasts [20] induced to undergo chondrogenic differentiation as 3D pellets for 21 days were used as a positive and negative control, respectively. Mounted pellets were scanned with a NanoZoomer S360 (Hamamatsu, Shizuoka, Japan) using OncoTopix Scan software (Visio Pharm, Hørsholm, Denmark) and NDP.view2 Plus (Hamamatsu) to view the images. Images of 5× magnification were captured and brightness and exposure settings for Safranin-O Redness analysis were kept constant. Images of the 0.1% Safranin-O stain were taken in a chamber slide as a pure red calibrator. Semiquantitative Redness analysis for proteoglycan content of each pellet was performed using Fiji software (ImageJ 1.49) as previously described [21]. In short, color contributions from the background light and microscope were corrected for by using the pure red calibrator and by subtracting the fixed background intensity measures from each individual histogram. After red balancing and background assessment, the mean red, green, and blue values were determined for all controls and pellets using the whole pellets as the focus area. The Redness value of each section was then calculated as follows:

$${Green}_{modified}=\left({Green}_{mean}-{Green}_{background}\right)$$

$${Blue}_{modified}={(Blue}_{mean}-{Blue}_{background})$$

$$Redness\;value={Red}_{mean}-\left(\frac{{Green}_{modified}+{Blue}_{modified}}{2}\right)$$

where *mean* represents the pellet value and *background* represents the pure red value. Redness values were normalized to articular cartilage and then averaged per cell line.

### 2.7. Osteogenic Differentiation

Passage 6 cells were seeded onto two 6-well plates (NUNC, Thermo Fisher Scientific) with a seeding density of 3000 cells/cm^2^. The cells were cultured in 2 mL expansion medium/well until 90% confluency where osteogenic induction was initiated, as previously described by our group [9]. Osteogenic induction medium consisted of DMEM (1 g/L glucose), 10% (*v*/*v*) FBS, 1% (*v*/*v*) P/S, 100 nM dexamethasone, 0.05 mM A2P, and 10 mM β-glycerophosphate disodium salt hydrate (Sigma-Aldrich). After 21 days of induction, the cells were assessed for calcium deposits using Alizarin Red S staining and for gene expression of osteogenic markers using qPCR. Non-induced cells grown in expansion medium for 21 days were used as a control.

### 2.8. Alizarin Red S

Osteogenic differentiation was assessed at day 21 with a colorimetric Alizarin Red S quantification kit (ARed-Q, ScienCell, Carlsbad, CA, USA) to detect calcium deposits following the manufacturer’s protocol [22] and as previously described [9]. Each cell line was analyzed in triplicate with 3 osteogenic-induced wells and 3 non-induced wells.

### 2.9. Gene Expression by qPCR

For chondrogenic assessment, RNA was harvested and isolated from 6 pellets after 21 days of chondrogenic induction and from 1 non-induced T75 monolayer at 70% confluence, as previously described [20]. For osteogenic assessment, RNA was harvested from 3 osteogenic-induced wells and from 3 non-induced control wells on day 21. RNA was harvested using QIAzol^®^ (Qiagen, Hilden, Germany), snap-frozen in liquid nitrogen, and stored at −80 °C before further isolation. For RNA isolation, the pellets were homogenized three times and the osteogenic-induced cultures were homogenized two times using a T10 basic Ultra-Turrax homogenizer (IKA, Thermo Fisher Scientific). Total RNA was isolated using Qiagen Rneasy Mini Kits^®^ (Qiagen) as per the manufacturer’s instructions. RNA yield was measured by using a Qubit BR Assay (Thermo Fisher Scientific) on a Qubit 3.0 fluorometer (Thermo Fisher Scientific) and a NanoDrop 1000 spectrophotometer (Thermo Fisher Scientific). All purified RNA samples had 260/280 and 260/230 ratios close to 2 on the Nanodrop, meeting RNA quality thresholds. Removal of potential DNA contamination and first-strand cDNA synthesis were carried out using a Maxima First Strand cDNA Synthesis Kit for qPCR with dsDNase (Thermo Fisher Scientific). Samples were diluted with nuclease-free water to 10.67 ng/µL cDNA (maximum concentration possible across samples for applied qPCR design). Steady-state mRNA levels were quantified using Taq-Man^®^ primer-probe sets (FAM-dye labeled MGB probes) (Thermo Fisher Scientific) (Table 1). A panel of 5 chondrogenic and 5 osteogenic biomarkers were selected for qPCR analysis together with 2 endogenous controls (GUSB and GAPDH). The endogenous controls were tested against a representative subset of experimental conditions and cell lines where NormFinder v21 software [23] identified GUSB to be most uniform across samples and was thus used for later analyses. Negative controls consisted of nuclease-free water, minus template, and minus master mix. All samples were run in duplicate with TaqMan^TM^ Fast Advanced Master Mix for qPCR (Thermo Fisher Scientific) on 96-well Roche PCR plates (Axygen, Sigma-Aldrich) using the Roche LightCycler^®^ 96 System (Roche, Basel, Switzerland). Data were analyzed with LightCycler^®^ 96 software version 1.1.0.1320, where cycle threshold (Ct) values were calculated. All primer-probes showed amplification efficiencies close to 2 when tested on serial dilutions except for the negative controls that showed no amplification. GUSB was used to calculate ΔCt values. Mean ΔCt values of induced unsorted cells were used as a calibrator to calculate ΔΔCt values. The relative expression (RQ) of biomarkers was calculated by RQ = 2^−ΔΔCt^. ln (RQ) was used for statistics to linearize the data [24].

### 2.10. Statistics

Normality of data was assessed by QQ plots and Shapiro–Wilk tests. If normally distributed, multiple paired *t*-tests were performed with Benjamini, Krieger, and Yekutieli post hoc correction. If the data were not normally distributed, multiple Wilcoxon matched-pairs signed rank tests were performed with Benjamini, Krieger, and Yekutieli post hoc correction. A simple linear regression and Pearson’s correlation test was applied to assess the correlation between CD271^+^ content and Safranin-O Redness value. *p*-values < 0.05 were considered statistically significant. All statistical analysis and graphical presentations were performed in GraphPad Prism version 10.1.2. The data are presented as the mean ± SD.

## 3. Results

### 3.1. Enrichment of CD34^+^, CD146^+^, and CD271^+^ MFAT-Derived Stem Cells by MACS

MACS resulted in an enrichment of CD34^+^, CD146^+^, and CD271^+^ stem cells with approximate doubling when assessed by flow cytometry (Figure 1). The prevalence of CD34^+^ stem cells (CD31^−^/CD34^+^/CD45^−^/CD90^+^/CD146^−^) was 45 ± 18% at passage 3 before MACS and 91 ± 14% at passage 6 after MACS. CD146^+^ stem cells (CD31^−^/CD34^−^/CD45^−^/CD90^+^/CD146^+^) went from 21 ± 10% before MACS to 50 ± 18% after MACS. CD271^+^ stem cells (CD31^−^/CD45^−^/CD90^+^/CD271^+^) went from 16 ± 6% before MACS to 34 ± 13% after MACS. Immunophenotyping for unsorted cells at passage 6 is shown in Figure 1C, where relatively stable immunophenotypes are seen when comparing unsorted cells at passage 3 and 6.

Before MACS, at passage 3, co-expression of CD34/CD146 (transitional pericytes) was detected in 5 ± 5%, CD34/CD271 were detected in 13 ± 7%, CD146/CD271 were detected in 2 ± 2%, and CD34/CD146/CD271 were detected in 1 ± 1% (Figure 1A). Of the CD34^+^ cells, 10 ± 7% co-expressed CD146, 18 ± 15% co-expressed CD271, and 1 ± 1% co-expressed CD146/CD271 after MACS. Of the CD146^+^ cells, 8 ± 3% co-expressed CD34, 3 ± 4% co-expressed CD271, and 2 ± 3% co-expressed CD34/CD271 after MACS (Figure 1B). Of the CD271^+^ cells, 13 ± 8% co-expressed CD34, 7 ± 9% co-expressed CD146, and 2 ± 3% co-expressed CD34/CD271 after MACS. Of the CD34^+^ stem cells (P6), 69% did not show co-expression of CD146 or CD271. Of the CD146^+^ stem cells (P6), 78% did not show co-expression of CD34 or CD271. Of the CD271^+^ stem cells (P6), 41% did not show co-expression of CD34 or CD146.

### 3.2. Chondrogenic Differentiation of MACS-Enriched MFAT-Derived Stem Cells 

#### No Effect of Cell Type on Chondrogenic Pellet Size

**Figure 2:**
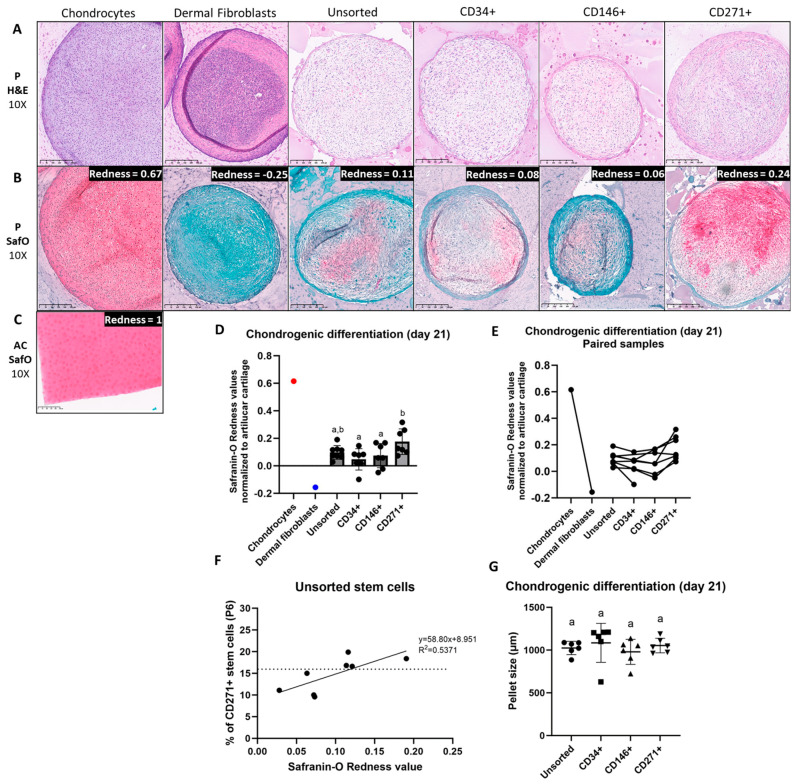
Histology and size of pellets (P) after 21 days of culture in chondrogenic-induced medium and articular cartilage. (**A**) Representative images of Hematoxylin and Eosin (H&E) stained pellets of chondrocytes, dermal fibroblasts, and unsorted, CD34^+^, CD146^+^, and CD271^+^ stem cells. (**B**) Representative images of Safranin-O (SafO)-stained pellets of chondrocytes (positive control), dermal fibroblasts (negative control), and unsorted, CD34^+^, CD146^+^, and CD271^+^ stem cells with Redness values normalized to articular cartilage. Proteoglycans are stained red/pink. (**C**) Representative image of articular cartilage (AC) stained with Safranin-O (calibrator for Redness analyses). Images are taken at 10× magnification. Size bars show 250 µm. (**D**) Proteoglycan Redness values of Safranin-O-stained pellets from chondrocytes (positive control), dermal fibroblasts (negative control), and unsorted, CD34^+^, CD146^+^, and CD271^+^ stem cells from microfragmented adipose tissue (MFAT) when normalized to articular cartilage. (**E**) Proteoglycan Redness values of Safranin-O-stained pellets showing paired samples. (**F**) Redness values of Safranin-O-stained pellets from unsorted stem cells as a function of CD271^+^ stem cell content with a linear regression model and Pearson’s correlation test (*p* = 0.04). Dotted line indicates the half with the highest and lowest CD271^+^ content and Redness values. (**G**) Dot plot of pellet size (µm) of chondrogenic induced unsorted, CD34^+^, CD146^+^, and CD271^+^ stem cells from MFAT. (**D**,**G**) Data are shown as mean with SD. Cell types not labeled with the same letter are statistically significantly different from each other (*p* < 0.05). (**D**) Data were normally distributed and analyzed by multiple paired *t*-tests. (**G**) Data were not normally distributed and analyzed using multiple Wilcoxon matched-pairs signed rank tests. (**F**) Data were normally distributed and analyzed with Pearson’s correlation test.

Chondrogenic pellet size (*n* = 6, N = 24) varied from 0.6 mm to 1.2 mm in diameter, as shown in Figure 2G. The pellet size of unsorted cells was 1025 ± 78 µm; of CD34^+^ stem cells, it was 1085 ± 228 µm; of CD146^+^ stem cells, it was 979 ± 147 µm; and of CD271^+^ stem cells, it was 1053 ± 86 µm. No effect of subtype on pellet size was seen when compared to unsorted cells (*p* > 0.4). No subtype showed larger chondrogenic pellet size than the other subtypes (*p* > 0.1).

### 3.3. Similar Chondrogenic Pellet Morphology Among MFAT-Derived Stem Cell Subtypes

H&E-labeled pellets from each stem cell type were assessed microscopically by a board-certified anatomic pathologist (JJ) with representative descriptions below. No relevant differences were identified among the analyzed stem cell subtypes. No major necrotic cellular center was identified in any of the stem cell pellets, which was seen in the negative dermal fibroblast control. Representative images of the H&E-labeled pellets are shown in Figure 2A.

Unsorted, CD34^+^, CD146^+^, and CD271^+^ stem cells: The pellets were composed of streams of spindle cells with minimal lightly basophilic cytoplasm and oval hyperchromatic nuclei embedded in a wispy eosinophilic stroma. Few cells exhibited mild pleomorphism. Scattered cells contained variably sized cytoplasmic vacuoles. A thin rim of spindle cells with moderate eosinophilic stroma bordered the pellets. Aggregates of eosinophilic material with or without faded pinpoint basophilic central material were interspersed. The pellets were of moderate cell density.

Chondrocyte pellets (positive control): The cell pellets were composed of well-differentiated spindle to stellate cells (chondrocytes) embedded in a lightly basophilic mucinous matrix.

Dermal fibroblast pellets (negative control): The cell pellets were largely composed of a densely cellular proliferation of polygonal cells with hyperchromatic nuclei (necrotic cells).

### 3.4. MFAT-Derived CD271^+^ Stem Cells Produced More Proteoglycans than the Other Subtypes, but Not More than Unsorted Stem Cells

Proteoglycans stained positively red after Safranin-O exposure, as seen in the positive controls of articular cartilage (calibrator) and chondrocyte pellets. Representative images of Safranin-O-labeled pellets and the Redness values normalized to articular cartilage are seen in Figure 2B,D. All stem cell pellets (except for three replicates; one CD34^+^ and two CD146^+^) showed proteoglycan formation. CD271^+^ stem cells produced more proteoglycans (0.18 ± 0.09 Redness) than CD34^+^ stem cells (0.05 ± 0.08 Redness) (*p* = 0.02) and CD146^+^ stem cells (0.07 ± 0.09 Redness) (*p* = 0.02), but not compared to unsorted stem cells (0.10 ± 0.05 Redness) (*p* = 0.07). Patients with the highest chondrogenic performance of unsorted cells also had the highest performance of CD271^+^ cells (Figure 2F). A positive correlation was identified between the Safranin-O Redness value and the level of CD271^+^ stem cells in unsorted stem cells from MFAT at passage 6 (*p* = 0.04) (Figure 2F). No redness was seen in the negative control of dermal fibroblasts. Control articular cartilage sections showed no difference between slide position.

### 3.5. Upregulation of Chondrogenic Biomarkers in MFAT-Derived Stem Cells

**Figure 3:**
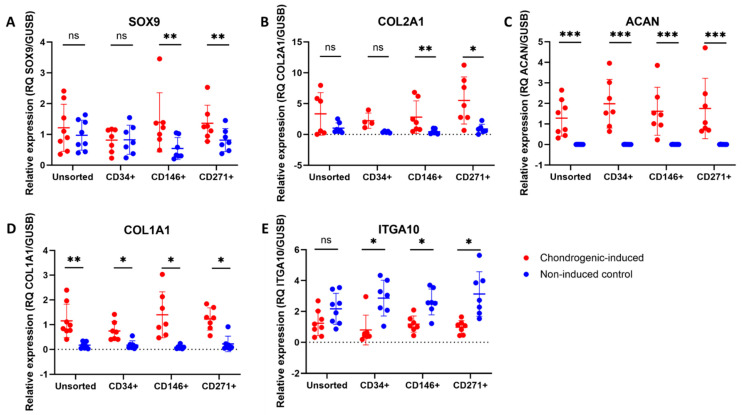
qPCR data of chondrogenic biomarkers of unsorted, CD34^+^, CD146^+^, and CD271^+^ stem cells from microfragmented adipose tissue. Dot plots showing the relative expression of (**A**) SOX9, (**B**) COL2A1, (**C**) ACAN, (**D**) COL1A1, and (**E**) ITGA10 of chondrogenic-induced pellets (day 21) and non-induced controls. Statistical comparisons are shown between induced and non-induced samples. ns: non-significant (*p* > 0.05), *: *p* < 0.05, **: *p* < 0.01, and ***: *p* < 0.001. The data are shown as the mean with SD. (**A**,**D**) The data were not normally distributed and analyzed using the Wilcoxon matched-pairs signed rank test. (**B**,**C**,**E**) The data were normally distributed and analyzed using multiple paired *t*-tests.

**Figure 4:**
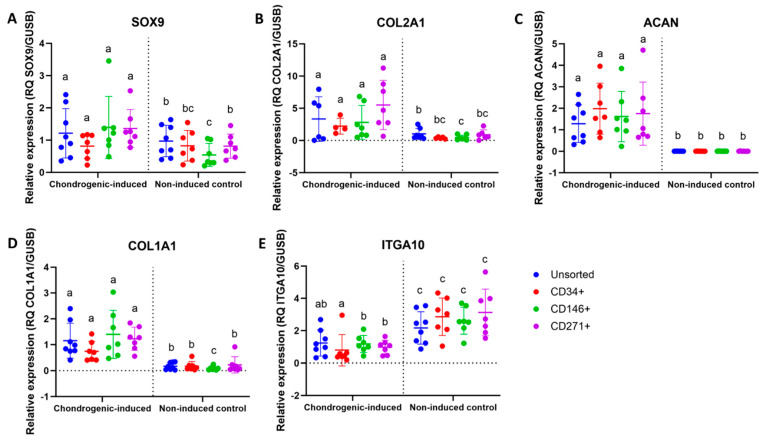
qPCR data of chondrogenic biomarkers of unsorted, CD34^+^, CD146^+^, and CD271^+^ stem cells from microfragmented adipose tissue. Dot plots showing the relative expression of (**A**) SOX9, (**B**) COL2A1, (**C**) ACAN, (**D**) COL1A1, and (**E**) ITGA10 of chondrogenic-induced pellets (day 21) and non-induced controls as a function of cell type. Statistical comparisons are shown between cell types. Within the same culture condition, cell types not labeled with the same letter are statistically significantly different from each other (*p* < 0.05). The data are shown as the mean with SD. (**A**,**D**) The data were not normally distributed and analyzed using the Wilcoxon matched-pairs signed rank test. (**B**,**C**,**E**) The data were normally distributed and analyzed using multiple paired *t*-tests.

An upregulation was seen in the steady-state mRNA expression of SOX9 and COL2A1 for CD146^+^ and CD271^+^ stem cells after chondrogenic induction, but not for CD34^+^ or unsorted stem cells. An upregulation of ACAN and COL1A1 was observed in all four cell types following chondrogenic induction. A downregulation of ITGA10 was seen for CD34^+^, CD146^+^, and CD271^+^ stem cells after chondrogenic induction, but not for unsorted stem cells. Differences between chondrogenic-induced and non-induced cells are shown in Figure 3. Differences between cell types are shown in Figure 4. For SOX9, COL2A1, ACAN, and COL1A1, no statistically significant differences were detected between cell types after chondrogenic induction. For ITGA10, chondrogenic-induced CD34^+^ stem cells had significantly lower gene expression compared to CD146^+^ and CD271^+^ stem cells, but not compared to unsorted stem cells. Gene expression of COL2A1 was higher than that of COL1A1 for all cell-type pellets, indicated by the overall lower Ct values of COL2A1 on qPCR.

### 3.6. Osteogenic Differentiation of MFAT-Derived Stem Cells

#### MFAT-Derived CD146^+^ Stem Cells Produced More Calcium Deposits than the Other Stem Cell Types

**Figure 5:**
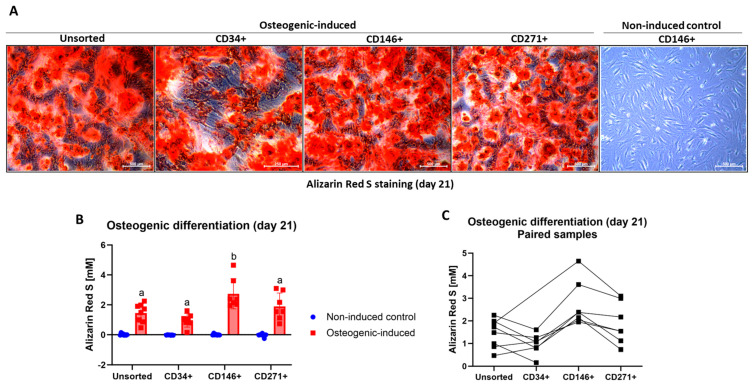
Alizarin Red S staining of osteogenic-induced stem cells on day 21. (**A**) Representative images of osteogenic-induced and non-induced stem cells after Alizarin Red S staining taken at 4× magnification. Size bars shows 500 µm. Calcium deposits are stained red. (**B**) Alizarin Red S concentration (mM) as a measurement of calcium deposits of osteogenic-induced and non-induced unsorted, CD34^+^, CD146^+^, and CD271^+^ stem cells from microfragmented adipose tissue. (**C**) Alizarin Red S values of osteogenic-induced samples shown as paired samples. (**B**) The data are shown as the mean with SD. Cell types within the same culture condition not labeled with the same letter are statistically significantly different from each other (*p* < 0.05). The data were not normally distributed and analyzed using multiple Wilcoxon matched-pairs signed rank tests.

Cellular expansion from plating on 6-well plates until 90% confluence and initiation of osteogenic differentiation took 13 ± 4 days for unsorted cells, 12 ± 4 days for CD34^+^ cells, 15 ± 7 days for CD146^+^ cells, and 15 ± 7 days for CD271^+^ cells. All MFAT-derived stem cell types were able to undergo osteogenic differentiation after 21 days of culture in osteogenic induction medium, as shown in Figure 5B. No calcium deposition was seen in non-induced control cultures. Alizarin Red S values of osteogenic-induced samples are shown as paired samples in Figure 5C. Representative images of osteogenic-induced and non-induced cells after Alizarin Red S staining on day 21 are shown in Figure 5A. Calcium deposition defined by Alizarin Red S concentration was statistically significantly higher in osteogenic-induced cells compared to non-induced controls (*p* ≤ 0.001). Osteogenic-induced CD146^+^ stem cells had a statistically significantly higher Alizarin Red S concentration than unsorted, CD34^+^, and CD271^+^ stem cells (*p* ≤ 0.03) (Figure 5B).

### 3.7. Upregulation of Some Osteogenic Biomarkers in MFAT-Derived Stem Cells

**Figure 6:**
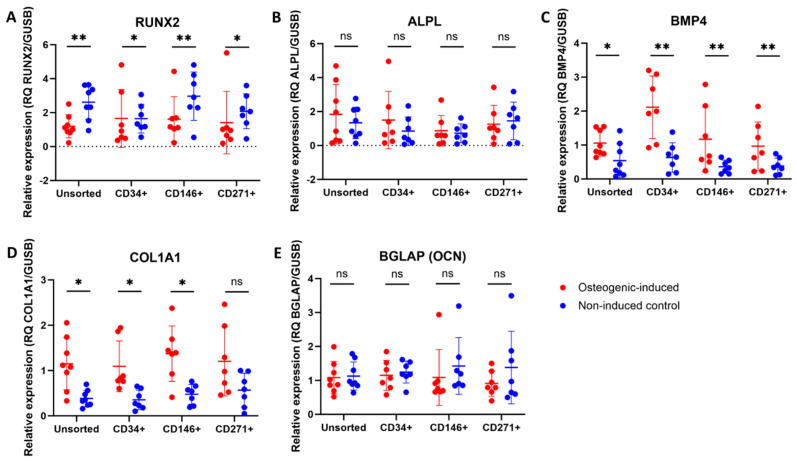
qPCR data of osteogenic biomarkers of unsorted, CD34^+^, CD146^+^, and CD271^+^ stem cells from microfragmented adipose tissue. Dot plots showing the relative expression of (**A**) RUNX2, (**B**) ALPL, (**C**) BMP4, (**D**) COL1A1, and (**E**) BGLAP (OCN) of osteogenic-induced monolayer cultures (day 21) and non-induced controls. Statistical comparisons are shown between induced and non-induced samples. ns: non-significant (*p* > 0.05), *: *p* < 0.05, and **: *p* < 0.01. The data are shown as the mean with SD. (**A**,**D**,**E**) The data were not normally distributed and analyzed using Wilcoxon matched-pairs signed rank tests. (**B**,**C**) The data were normally distributed and analyzed using multiple paired *t*-tests.

**Figure 7:**
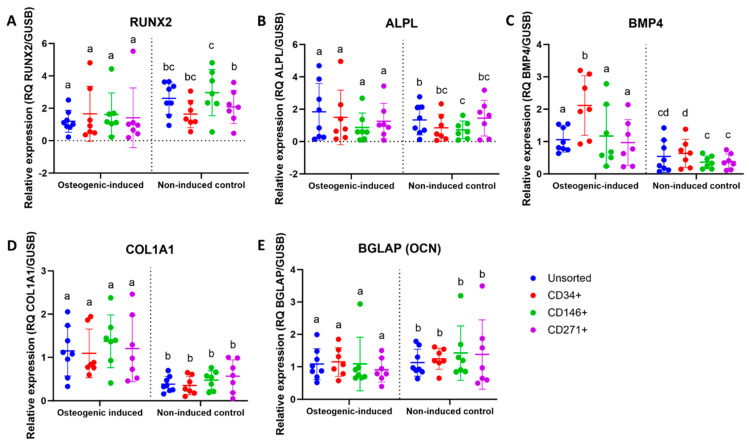
qPCR data of osteogenic biomarkers of unsorted, CD34^+^, CD146^+^, and CD271^+^ stem cells from microfragmented adipose tissue. Dot plots showing the relative expression of (**A**) RUNX2, (**B**) ALPL, (**C**) BMP4, (**D**) COL1A1, and (**E**) BGLAP (OCN) of osteogenic-induced monolayer cultures (day 21) and non-induced controls as a function of cell type. Statistical comparisons are shown between cell types. Within the same culture condition, cell types not labeled with the same letter are statistically significantly different from each other (*p* < 0.05). The data are shown as the mean with SD. (**A**,**D**,**E**) The data were not normally distributed and analyzed using the Wilcoxon matched-pairs signed rank test. (**B**,**C**) The data were normally distributed and analyzed using multiple paired *t*-tests.

An upregulation was seen in the steady-state mRNA expression of BMP4 and COL1A1 for all cell types after 21 days of osteogenic induction when compared to non-induced controls. A downregulation of RUNX2 was seen in all cell types after osteogenic induction for 21 days. No statistically significant differences were seen in gene expression of ALPL or BGLAP (OCN) between osteogenic-induced and non-induced controls on day 21. Differences between osteogenic-induced and non-induced controls are shown in Figure 6. Differences between cell types are shown in Figure 7. For RUNX2, ALPL, COL1A1, and BGLAP (OCN), no statistically significant differences were detected between cell types after osteogenic induction. For BMP4, osteogenic-induced CD34^+^ stem cells showed significantly higher gene expression compared to unsorted, CD146^+^, and CD271^+^ stem cells.

## 4. Discussion

The present study showed that the enrichment of CD34^+^, CD146^+^, and CD271^+^ stem cells from MFAT was feasible using MACS and that all subtypes were able to undergo chondrogenic and osteogenic differentiation, with some cell type differences.

### 4.1. Enrichment of MFAT-Derived Stem Cell Subtypes

The enrichment of CD34^+^, CD146^+^, and CD271^+^ MFAT-derived stem cells with an approximate doubling corresponds well with a previous study in which Herrmann et al., (2016) identified 44.1 ± 7.4% CD146^+^ stem cells before MACS and 76.5 ± 7% after MACS when derived from the stromal vascular fraction (SVF) of adipose tissue [17]. Greater enrichment was reported by Li et al., (2019), where CD146^+^ cells from SVF increased from 13.48% to 88.12% after MACS, but the description of the enrichment procedure lacks sufficient detail for reproducibility [31]. Lauvrud et al., (2017) reported 18.85 ± 3.1% CD146^+^ stem cells in SVF after MACS but did not report the level before MACS [32]. In the present study, further enrichment of CD271^+^ cells was attempted by sorting the isolated fraction from the first sorting into a second column. Unfortunately, this resulted in low cell viability and was therefore not applied in the study. In the current study, immunophenotyping was not performed directly after MACS, but at passage 6 just prior to the differentiation assays, to allow the cells to recover after sorting, to ensure enough cells for further expansion and differentiation assays, and to provide knowledge on the immunophenotype of the cells being assessed for differentiation capacity.

Co-expression of CD34, CD146, and/or CD271 was observed after MACS by flow cytometry, resulting in some cell types appearing in multiple cell groups. This is a limiting factor but is believed to be of minor importance as most cells in each isolated cell subtype fraction did not show co-expression. Nevertheless, half of the CD271^+^ cells showed co-expression of CD34 or CD146. A comparison of CD271^+^ cells with and without co-expression would be interesting for future studies.

A higher sorting efficiency and less co-expression could be obtained using fluorescence-activated cell sorting (FACS), as shown previously [14,33], where CD271^+^ stem cells from SVF were enriched from 21.8 ± 6.4% to 97.8 ± 1.1% after FACS [14]. The current FACS modalities are, however, associated with a risk of cell contamination from other samples, as the intra-machine cellular materials are not sterile or for single use. This is particularly problematic when working with relatively slow proliferating stem cells, as faster proliferating contaminant cells can become substantial and bias the results. FACS was therefore deemed unsuitable for the current study, as the cells had to be cultured after sorting and because we aimed to perform clinically relevant research from an aseptic perspective. Future research should explore the sorting efficiency of new cell sorting methods aimed at clinical use and their cost-effectiveness.

### 4.2. Chondrogenic Potential of MFAT-Derived Stem Cells

The results showed that unsorted, CD34^+^, CD146^+^, and CD271^+^ stem cells from MFAT were able to perform in vitro chondrogenic differentiation when using a 3D model. This stands in contrast to previous 3D studies in which stem cells derived from enzymatically digested adipose tissue (SVF) did not show chondrogenic differentiation performance and resembled the potential of dermal fibroblasts as a negative control [17,20,21]. This might be due to differences in the cellular composition of MFAT and SVF, where MFAT has a higher content of pericytes [9,10,34]. Another study on MFAT-derived unsorted stem cells showed some chondrogenic differentiation potential when assessed in a 2D model [35]. Three-dimensional models are, however, reported to be most suitable for chondrogenic differentiation and to best simulate in vivo articular cartilage conditions with hypoxia in the center of the pellet [36,37]. The chondrogenic upregulation of proteoglycans seen at the protein level through Safranin-O staining was supported by our qPCR data, which showed that all cell types showed an upregulation in ACAN after chondrogenic induction, as aggrecan is an essential proteoglycan in articular cartilage [2].

Our data showed that CD271^+^ stem cells from MFAT have a higher potential for generating proteoglycans in vitro under standard chondrogenic conditions compared to CD34^+^ and CD146^+^ stem cells, but not compared to unsorted stem cells from MFAT. This is supported by a previous study by Kohli et al., (2019) in which CD271^+^ stem cells from SVF showed no difference in chondrogenic in vitro differentiation performance when compared to unsorted cells but did show enhanced cartilage repair with reduced vascularization in a rat osteochondral wound model when compared to unsorted stem cells [15]. As shown in Figure 2F, patients with the highest chondrogenic performance of unsorted cells also exhibited the highest performance of CD271^+^ cells. These patients were in the top half with the highest content of CD271^+^ stem cells, both before and after MACS. CD271^+^ stem cells from MFAT therefore appear to be a promising candidate for direct cartilage regeneration. It is likely that greater CD271^+^ sorting efficiency and purity would result in higher chondrogenic performance, potentially also compared to unsorted cells. More research is needed to elucidate if cell signaling between heterogenous cells is advantageous compared to a greater purified CD271^+^ population. The higher extracellular proteoglycan content in CD271^+^ stem cells was surprisingly not reflected in pellet size; however, the standard deviation was narrower than that observed for CD34^+^ and CD146^+^ stem cells, reflecting a more uniform “large” pellet size. A lack of correlation between pellet size and proteoglycans has previously been reported [20].

From a morphological perspective, the low number of necrotic cells in the center of the stem cell pellets was also interesting as it suggests a cellular adaptation to low oxygen levels, which stands in contrast to the dermal fibroblast pellets with a large necrotic center. Such a characteristic would be important, as articular chondrocytes live in vivo under hypoxic conditions with low oxygen levels between 1 and 10% depending on the distance to the articular surface [38].

All of the stem cell types had greater gene expression of COL2A1 compared to COL1A1. Gene expression of COL1A1 is reported in stem cell pellets after 21 days of chondrogenic culture but is considered unwanted since COL1A1 is a biomarker of biomechanically inferior fibrocartilage when compared to hyaline cartilage abundant in COL2A1 [20,39]. When assessing early (SOX9) and late biomarkers (COL2A1 and ACAN) of chondrogenesis, CD146^+^ and CD271^+^ stem cells showed the greatest difference between induced and non-induced cells. ITGA10 was downregulated in CD34^+^, CD146^+^, and CD271^+^ stem cells after chondrogenic induction. This may occur as ITGA10 is a biomarker relevant for collagen type II binding, which may be more essential for the homing of undifferentiated cells than for chondrocyte differentiated cells. Nevertheless, human integrin α10β1-marked MSCs have been identified in cartilage defects and exhibit a chondrocyte-like phenotype [25]. The mean expression of ITGA10 was highest in undifferentiated CD271^+^ stem cells, although not statistically significant different from the other cell types.

### 4.3. Osteogenic Potential of MFAT-Derived Stem Cells

The results show that unsorted, CD34^+^, CD146^+^, and CD271^+^ stem cells from MFAT were able to perform in vitro osteogenic differentiation and that CD146^+^ stem cells had the highest osteogenic potential when measured by gold-standard Alizarin Red S calcium staining. This is supported by previous studies on SVF [13,14,20] in which pericytes/CD146^+^ stem cells have shown high orthopedic performance both in vitro and in animal models [11,13,16,17,33]. Nevertheless, Wang et al., (2019) showed that pericytes co-cultured with adventitial stem cells from SVF had greater osteogenic in vitro differentiation performance than when cultured alone. The same study also showed a synergistic effect of CD34^+^ and CD146^+^ stem cells from SVF on bone healing in a calvarian defect mouse model, compared to either cell type alone [16]. In addition, James et al., (2012) reported greater healing of critical-size calvarian defects in a mouse model when using a combination of CD146^+^ and CD34^+^ stem cells compared to unsorted SVF, but the study did not investigate each subtype alone [40]. The synergistic effect of a mixed cell population was not supported by the current study, in which CD146^+^ stem cells showed greater osteogenic differentiation compared to unsorted stem cells. Whether a combination of purified CD34^+^ and CD146^+^ cells from MFAT would improve osteogenesis remains to be investigated. Due to the higher content of CD146^+^ stem cells in MFAT compared to SVF [9,10], it would be interesting to investigate MFAT further for treatment of bone lesions, potentially after CD146^+^ enrichment. Promising bone healing results are shown in a dog model using MFAT [41].

Alizarin Red S staining of calcium deposits was used as a gold standard to verify osteogenic differentiation [22]. The clear calcium deposition identified both macroscopically and through spectrophotometry was surprisingly not well reflected in the qPCR data, except for BMP4 and COL1A1. This may be explained by the timing of RNA harvest on day 21 of osteogenic induction, which is generally standard and made to match the timing of Alizarin Red S staining. Earlier and later timepoints have shown lower gene expression of medium-to-late osteogenic biomarkers and calcium deposition [9,22,42,43]. RUNX2 and ALPL are both early markers of osteogenesis. RUNX2 is a transcription factor essential for the activation of osteoblast-associated genes like ALPL and OCN [27]. ALPL is highly expressed in mineralized tissue where it enzymatically degrades inhibitory pyrophosphates that bind calcium to enhance mineralization. Importantly, the highest ALPL gene expression and protein activity level is reported 6 to 7 days after initiation of osteogenic induction [28,30,44]. Therefore, it is likely that higher expression levels of RUNX2 and ALPL would have been observed in the induced samples if the RNA had been harvested at earlier timepoints. In addition, alkaline phosphatase activity could have been measured on protein level on day 7 of osteogenic differentiation [20]. On the other hand, OCN is primarily produced by mature osteoblasts during mineralization [30], which may explain the low levels identified on day 21 of osteogenic induction. Thus, it is possible that higher gene expression of OCN would be seen during longer culture periods in osteogenic induction medium, as shown previously [44,45]. In support of our data, BMP4 is secreted by (early) osteoblasts and is a highly potent inducer of bone mineralization and collagen fiber formation. Consequently, BMP4 and COL1A1 are found in osteoblasts of developing bone and in osteogenic-induced adipose tissue-derived stem cells [29,46].

### 4.4. General Discussion

The paired-sample design with cell lines from the same OA patient and rigorous use of controls are clear strengths of the current study. The use of MFAT from OA patients heightens the translational clinical value of autologous cells compared to studies using samples from non-OA donors [14,15,16], although inclusion of more patients would strengthen the generalizability. Ideally, assessments would have been performed immediately after harvest to best simulate MFAT treatment. This was, however, not feasible due to the number of cells required for the assays. Passage 6 was therefore the earliest passage number possible to perform all of the described assays. However, this is believed to be a minor bias as the cellular composition was relatively stable throughout cell expansion (Figure 1C), and previous studies have shown that adipose tissue-derived stem cells are stable at least until passage 7–9 [47,48]. The chondrogenic pellet size was measured in 2D to maintain the aseptic environment essential for later qPCR analyses. Two-dimensional images were taken of 10 pellets per cell line (10 technical replicates) to lessen the bias of the pellets being in the same orientation, as the pellets did not always have a fully circular three-dimensional shape.

We investigated direct differentiation performance in culture, even though paracrine signaling has been identified as the primary mechanism for clinical efficacy [10,49]. However, assessing the direct differentiation capacity of isolated stem cell subtypes is important, as growth factor secretion has been positively linked to differentiation capacity [50]. Differentiation capacity is also becoming increasingly important when applied together with scaffolds that enhance stem cell engraftment and direct differentiation at the injury site to improve tissue regeneration [16,31,51].

The results show that CD146^+^ and CD271^+^ stem cells have the greatest osteogenic and chondrogenic potential for calcium and proteoglycan deposition, respectively. As OA affects both the subchondral bone and articular cartilage, combined therapy might be beneficial. It was, however, interesting that CD271^+^ stem cells from MFAT exhibited both a relatively high chondrogenic and osteogenic differentiation capacity. As articular cartilage is more difficult to regenerate than subchondral bone in OA [2,52,53], CD271^+^ stem cells would be a promising candidate for further purification with newly developed aseptic sorting machines for further clinical testing. The potency of CD271^+^ stem cells from MFAT is supported by Beckenkamp et al., (2018) who identified CD271^+^ cells from SVF to have a better osteogenic differentiation performance than CD271- stem cells [14]. In addition, Kohli et al., (2019) showed that CD271^+^ cells from SVF improved early-stage osteochondral repair in rats [15].

Further in vivo studies are needed to elucidate the optimal stem cell composition and to define cut-offs for the content of potent cells (especially CD146^+^ and CD271^+^) for the improvement in clinical efficacy. A suggested threshold for further investigation of MFAT treatment in OA patients is a stem cell composition containing more than 15% CD271^+^ stem cells, as these cell lines exhibited some of the highest chondrogenic potential within the unsorted stem cell population, as shown in Figure 2F. This knowledge would enable the selection of suitable patients for MFAT treatment in a personalized manner based on a small adipose tissue biopsy. Patient and subtype selection should be guided by the indication and the affected tissue(s).

## 5. Conclusions

CD146^+^ stem cells showed the greatest osteogenic differentiation performance for calcium deposition, while CD271^+^ stem cells showed the greatest chondrogenic differentiation performance for proteoglycan formation. The prevalence of these stem cell types may be critical to clinical effectiveness when treating OA.

## Figures and Tables

**Table 1:** Overview of TaqMan primer-probes used for qPCR.

Panel	Gene	Gene Name(s)	Annotation	Human Assay ID
**Chondrogenic**	SOX9	SRY-box transcription factor 9	Early chondrogenic marker [2]	Hs00165814_m1
	ACAN	Aggrecan	Proteoglycan found in articular cartilage [2]	Hs00153936_m1
	COL2A1	Collagen type 2 alpha 1	Specific marker of articular hyaline cartilage [2]	Hs00264051_m1
	ITGA10	Integrin subunit alpha 10	A collagen type II-binding integrin expressed in cartilage tissue [25]	Hs01006910_m1
	COL1A1	Collagen type 1 alpha 1	High in fibrocartilage [26]	Hs00164004_m1
**Osteogenic**	RUNX2	Runt-related transcription factor 2	Early osteogenic transcription factor; master switch for activation of osteoblasts [27]	Hs01047973_m1
	ALPL	Alkaline phosphatase	Early marker of Osteogenesis; high levels in osteoblasts [28]	Hs01029144_m1
	BMP4	Bone morphogenic factor 4	Involved in bone mineralization [29]	Hs01041266_m1
	COL1A1	Collagen type 1 alpha 1	Major protein component of bone extracellular matrix [30]	Hs00164004_m1
	BGLAP (OCN)	Osteocalcin, bone gamma- Carboxyglutamic acid- containing protein	Secreted by mature osteoblasts; essential for bone mineralization [30]	Hs01587814_g1
**Endogenous controls**	GUSB	Glucuronidase-beta		Hs00939627_m1
	GAPDH	Glyceraldehyde-3-phosphate dehydrogenase		Hs02786624_g1

## Data Availability

The original contributions presented in this study are included in the article; further inquiries can be directed to the corresponding author.
